# Vascular endothelial growth factor-D is an independent prognostic factor in epithelial ovarian carcinoma

**DOI:** 10.1038/sj.bjc.6600701

**Published:** 2003-01-28

**Authors:** Y Yokoyama, D S Charnock-Jones, D Licence, A Yanaihara, J M Hastings, C M Holland, M Emoto, M Umemoto, T Sakamoto, S Sato, H Mizunuma, S K Smith

**Affiliations:** 1 Reproductive Molecular Research Group, Department of Obstetrics and Gynaecology, University of Cambridge, The Rosie Maternity Hospital, Robinson Way, Cambridge CB2 2SW, UK; 2 Department of Obstetrics and Gynecology, Hirosaki University School of Medicine, 5-Zaifu-cho, Hirosaki 036-8562, Japan

**Keywords:** VEGF-C, VEGF-D, VEGFR-3, epithelial ovarian carcinoma, prognosis, lymph node metastasis

## Abstract

We assessed the presence of vascular endothelial growth factor (VEGF)-C, VEGF-D and their receptor VEGFR-3 by immunohistochemistry in 59 epithelial ovarian carcinomas, 11 borderline tumours and 20 benign cystadenomas. VEGF-C and VEGF-D were generally expressed in tumour cells and also in endothelia adjacent to tumour nests which showed a strong staining for them. VEGFR-3 was expressed in lymphatic and vascular endothelial cells adjacent to tumour nests. Immunoreactivity was significantly more frequent as lesions progressed from a benign tumour to advanced carcinoma. A strong correlation was found between VEGF-C and VEGF-D detected in carcinoma and VEGFR-3 detected in neighbouring endothelial cells. Increased expression of VEGF-C, VEGF-D and VEGFR-3 was significantly associated with lymph node metastasis and peritoneal metastasis outside the pelvis. There was a significant correlation between the high levels of VEGF-C and VEGF-D proteins, and poor survival. The presence of VEGF-D was an independent prognostic indicator by multivariate analysis. We conclude that VEGF-C, VEGF-D and VEGFR-3 play an important role in lymphatic spread and intraperitoneal tumour development in ovarian carcinoma. Since VEGF-D was found to be an independent predictor of poor outcome, its measurement, together with other prognostic markers may improve prospective identification of patients with a poor prognosis.

Survival rates for patients with epithelial ovarian carcinoma have shown modest improvement in the past decade, but remain unsatisfactory (Yokoyama *et al*, 1999). Ovarian carcinoma has the highest mortality rate among gynaecological malignancies. When the disease is limited to the ovaries, the 5-year survival rate is more than 90%, whereas when it has extended to the intra-abdominal or retroperitoneal cavity, the survival rate decreases to only 30% (Greenlee *et al*, 2000). Established prognostic parameters for epithelial ovarian carcinomas include surgical staging, histological subtype, grading, lymph node metastasis and residual tumour after cytoreductive surgery (Yokoyama *et al*, 1999), but there is a lack of clinically reliable molecular markers for assessing prognosis in ovarian carcinoma. Thus, it is urgently required to identify additional prognostic parameters and to clarify the mechanism by which tumour spreads from the ovaries to distant sites.

It is well established that angiogenesis, the formation of new blood vessels, is necessary for the growth and metastatic spread of solid tumours (Folkman, 1990). Tumour progression is regulated by numerous stimulators and inhibitors of tumour angiogenesis (Folkman, 1995). Among angiogenic stimulators, vascular endothelial growth factor (VEGF)-A plays essential roles in vasculogenesis and angiogenesis (Beck and D'Amore, 1997), and plays a crucial role in tumour angiogenesis in a variety of carcinomas (Ellis and Fidler, 1996). However, the significant roles of other VEGF family members, VEGF-B, VEGF-C, VEGF-D and placenta growth factor (PLGF) in tumour angiogenesis and metastasis are not fully known (Nicosia, 1998).

VEGF-C stimulates the proliferation of both vascular and lymphatic endothelial cells *in vitro* (Joukov *et al*, 1997; Pepper *et al*, 1998), and promotes angiogenesis or hyperplasia of lymphatic vessels *in vivo* (Jeltsch *et al*, 1997; Cao *et al*, 1998; Witzenbichler *et al*, 1998) via VEGF receptor (VEGFR)-2 (KDR) and VEGFR-3 (Flt-4). VEGFR-2 is predominantly expressed by activated endothelia of blood vessels (Joukov *et al*, 1996; Neufeld *et al*, 1999) and VEGFR-3 is predominantly expressed by lymphatic endothelia (Kaipainen *et al*, 1995; Jussila *et al*, 1998). Increased levels of VEGFR-3 have been detected in lymphatic endothelia adjacent to carcinoma cells and in lymph nodes containing carcinoma metastases (Kaipainen *et al*, 1995; Jussila *et al*, 1998). VEGF-D, known as c*-fos*-induced growth factor (Orlandini *et al*, 1996), also binds to and activates VEGFR-2 and VEGFR-3 (Achen *et al*, 1998; Baldwin *et al*, 2001), suggesting a role for VEGF-D in tumour angiogenesis and lymphangiogenesis. Recent work has provided direct evidence that VEGF-C and VEGF-D are not only important regulators of tumour-induced lymphangiogenesis, but also enhance lymphatic metastasis in mouse tumour models (Mandriota *et al*, 2001; Skobe *et al*, 2001; Stacker *et al*,2001). A soluble form of VEGFR-3 has been found to be a potent inhibitor of VEGF-C and VEGF-D signalling when expressed in the skin of transgenic mice (Makinen *et al*, 2001).

Identification of a correlation between the presence of VEGF-C and VEGF-D and patient survival as well as lymphatic metastasis in different carcinoma types might lead to a novel therapeutic approach to prevent tumour progression in many carcinomas.

In this retrospective study, we assessed the presence of VEGF-C, VEGF-D and VEGFR-3 in benign tumours, borderline tumours and carcinomas of the ovary by immunohistochemistry. We determined their relation to known prognostic factors for ovarian carcinoma.

## Materials and methods

### Study population and tissues

Immunohistochemical examination was performed retrospectively on 90 epithelial ovarian tumours obtained from women who were surgically treated at the Hirosaki University Hospital between 1989 and 2000 after informed consent had been obtained. The tissue specimens included 59 epithelial ovarian carcinomas, 11 borderline tumours and 20 benign cystadenomas. All patients with epithelial ovarian carcinoma were surgically staged in accordance with the 1988 International Federation of Gynaecology and Obstetrics (FIGO) criteria. Namely, they underwent total hysterectomy, bilateral salpingo-oophorectomy, partial omentectomy, appendectomy, and pelvic and para-aortic lymphadenectomies. Patients included in this study had not received any preoperative chemotherapy. The breakdown for stages of ovarian carcinomas consisted of 27 patients with stage I, six with stage II, 19 with stage III and seven with stage IV. Histological types were classified into 32 cases with serous cystadenocarcinoma, seven with mucinous cystadenocarcinoma, 12 with endometrioid adenocarcinoma, six with clear cell adenocarcinoma, and two with undifferentiated adenocarcinoma. All cases were re-evaluated for histological type and grade by the same gynaecological pathologist (SS). All patients with ovarian carcinoma received postoperative chemotherapy combining cisplatin (60 mgm^−2^), epirubicin (40 mg m^−2^) and cyclophosphamide (300 mgm^−2^). The duration of follow-up ranged from 8 to 156 months (median, 54 months). During that median follow-up period, there were 20 carcinoma-specific deaths. The mean age of patients with ovarian carcinoma at surgery was 54.1 years (range, 28–78 years). Of the 11 borderline tumours, six were serous, and five mucinous. Of the 20 benign cystadenomas, 12 were serous, and eight mucinous. Patients with benign or borderline tumours did not receive any postoperative chemo-therapy and all of them remained alive at the end of the study.

### Immunohistochemistry

Goat polyclonal anti-VEGF-C (Cat No. AF752), anti-VEGF-D (Cat No. AF286) and anti-VEGFR-3 (Cat No. AF349) antibodies were purchased from R&D Systems (Abingdon, UK). Anti-VEGF-C, anti-VEGF-D and anti-VEGFR-3 antibodies were used at a concentration of 5, 5 and 1 *μ*g ml^−1^, respectively. All samples surgically obtained for immunohistochemistry were immediately fixed in formaldehyde and embedded in paraffin wax. Tissue sections (6 *μ*m) were passed through xylene and graded alcohols. Sections were placed in 0.01 M citrate buffer, pH 6.0, and heated at 800 W in a microwave oven for 5 min to retrieve tissue antigen. The sections were treated with 0.3% hydrogen peroxide (H_2_O_2_) in methanol for 10 min to quench the endogenous peroxidase activity within the tissue. Nonspecific binding sites were blocked with 1% BSA and 20% heat-inactivated rabbit serum in phosphate-buffered saline (PBS) for 30 min at room temperature (RT). The sections were incubated overnight at 4°C in the presence of the primary antibody. Then slides were washed (three changes over 1 h) in PBS containing 0.1% Tween 20 (PBS/Tween), before the application of the secondary biotinylated antibody (DAKO, Kyoto, Japan). Tissue was incubated with secondary antibody for 1 h at RT before being washed for 15 min with three changes of PBS/Tween. The sections were then incubated for 30 min with avidin-biotinylated–horseradish peroxidase complex (Vectastain Elite ABC kit, Vector Lab., Peterborough, UK) and reactivity visualised with 0.02% 3,3′-diaminobenzidine tetrahydrochloride (DAB, Sigma) as a chromogen in Tris-HCl buffer (pH 7.6) containing 0.03% H_2_O_2_. The sections were counterstained with Mayer's Haemalum. For each antibody, negative control studies were performed in which normal goat serum was used instead of the primary antibody. No significant staining was observed in the negative control sections. As positive controls, formalin-fixed paraffin-embedded sections from normal human placenta were stained for VEGF-C and VEGF-D, and paraffin sections from human endometrial adenocarcinoma known to express VEGFR-3 (Yokoyama *et al*, 2000) were stained by the same procedure. Two observers independently evaluated and interpreted the results of immunohistochemical staining, without knowledge of the clinical data of each patient. VEGF-C and VEGF-D staining were assessed by estimating the percentage of tumour cells in which staining was as intense as that of positive control cells or more intense than it and placing tumours into four groups: −(0%), negative; ±(<10%), weak; +(10–50%), moderate; ++(>50%), strong. Faint or equivocal immunoreaction was ignored. Tumours were considered positive when they showed moderate or strong staining for VEGF-C and VEGF-D (Umemoto *et al*, 2001). Regarding VEGFR-3 staining, cases in which at least 5% of endothelial cells adjacent to tumour nests were as strongly immunoreactive as positive control cells were considered positive (Kajita *et al*, 2001). We also carried out immunostaining on the serial sections using anti-CD31 antibody (R&D Systems, Abingdon, UK, Cat No. BBA7) to distinguish lymphatic from vascular endothelium. We detected VEGFR-3 expression on both lymphatic and vascular endothelia neighbouring tumour nests.

### Statistical analysis

The statistical significance of the relation between detection of VEGF-C, VEGF-D and VEGFR-3 and clinico-pathological factors was evaluated with univariate analysis using *χ*^2^ test and Fisher's exact probability test. Carcinoma-specific survival rates were calculated by the Kaplan–Meier method, and the statistical significance of differences in the cumulative survival curves between the groups was evaluated by the log-rank test. Multivariate survival analysis was performed using Cox's propor tional hazard method. Other statistical analysis was carried out using the Mann–Whitney *U*-test. A result was deemed significant at *P*<0.05.

## Results

### Detection of VEGF-C and VEGF-D in ovarian tumours, and detection of VEGFR-3 in endothelial cells adjacent to tumour nests

The frequencies of detection of VEGF-C and VEGF-D in ovarian tumours and those of VEGFR-3 in endothelial cells adjacent to tumour nests are demonstrated in Table 1

**Table 1:** Detection of VEGF-C, VEGF-D and VEGFR-3 in ovarian tumours

**Tissues**	**No. of patients**	**VEGF-C detection (%)**	**VEGF-D detection (%)**	**VEGFR-3 detection (EC) (%)**							
		0	<10%	10–50%	50% <	0	<10%	10–50%	50% <	Negative	Positive
Benign tumour	20	12(60.0)	8(40.0)	0	0	14(70.0)	6(30.0)	0	0	18(90.0)	2(10.0)
Borderline tumour	11	4(36.4)	5(45.4)	2(18.2)	0	6(54.5)	3(27.3)	2(18.2)	0	8(72.7)	3(27.3)
Carcinoma	59	0	20(33.9)	17(28.8)	22(37.3)	4(6.8)	18(30.5)	22(37.3)	15(25.4)	25(42.4)	34(57.6)

The incidence of VEGF-C and VEGF-D detected in carcinomas was significantly higher than that detected in benign tumours (Mann–Whitney *U*-test, *P*<0.0001, respectively) and borderline tumours (Mann–Whitney *U*-test, *P*=0.0002 and 0.0007, respectively). The frequency of VEGFR-3 detected in carcinomas was significantly higher than that detected in benign tumours (*χ*^2^ *P*<0.0002) and high with a marginal significance compared to that detected in borderline tumours (*χ*^2^, *P*=0.06). There was no significant difference in the levels of VEGF-C, VEGF-D and VEGFR-3 positivity between samples of benign and borderline tumours.

. In benign tumours, tumour cells failed to stain or showed very weak staining for VEGF-C and VEGF-D. Approximately 80% of borderline tumours failed to stain or showed weak staining for VEGF-C and VEGF-D, while a few cases showed moderate staining. Correspondingly, 90 and 72% of benign and borderline tumours failed to stain for VEGFR-3 in endothelial cells adjacent to tumour cells, respectively. In ovarian carcinomas, 66 and 63% of cases showed moderate or strong staining for VEGF-C and VEGF-D, respectively. In all, 57% of ovarian carcinomas stained positively for VEGFR-3 in endothelial cells adjacent to the carcinoma.

**Figure 1:**
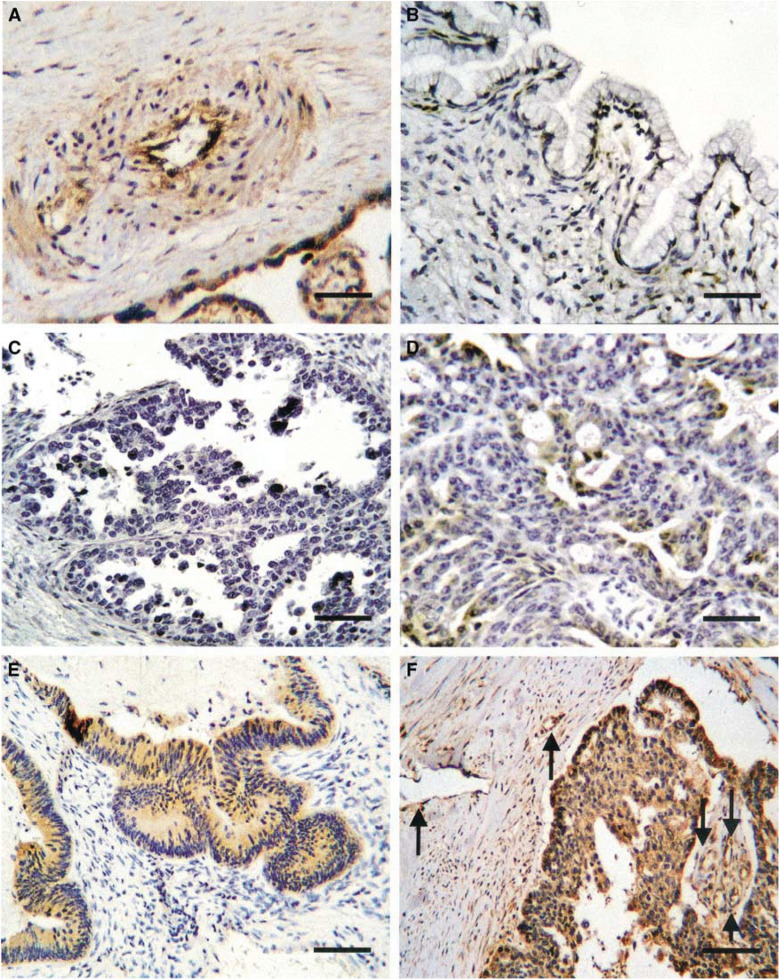
Immunohistochemical staining of VEGF-D in ovarian tumours and positive control. (**A**) Positive control in placental vessel and villi (scale bar, 25 mm). (**B**) Negative staining in benign cystadenoma (scale bar, 25 mm). (**C**, **D**, **E**, and **F**) −: negative, ±: weak, +: moderate, ++: strong, respectively, in epithelial ovarian carcinoma tissues (scale bar in **C** and **D**: 25 mm, scale bar in **E** and **F**: 50 mm). Arrows in **F** show VEGF-D-positive lymphatic and vascular endothelia adjacent to the carcinoma.

**Figure 2:**
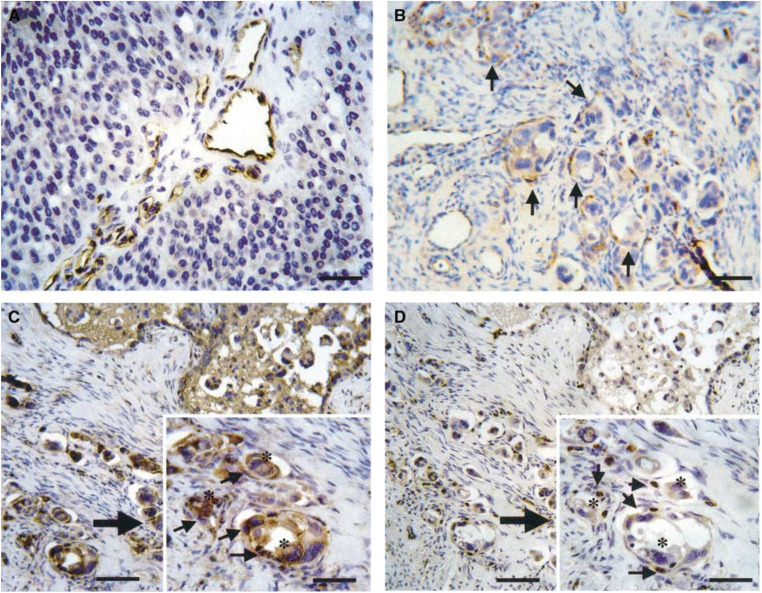
Immunohistochemical staining of VEGFR-3 and VEGF-D in endothelial cells adjacent to carcinoma. (**A**) VEGFR-3-positive lymphatic and vascular endothelial cells (scale bar, 25 mm). (**B**) VEGFR-3 staining highlighted vessel invasion by carcinoma cells (arrows) (scale bar, 25 mm). VEGF-D-positive endothelial cells (**C**) in vessels invaded by ovarian carcinoma was positive for VEGFR-3 (**D**) (scale bars 25 mm). Arrows on each inset show endothelia that were positive for VEGF-D (**C**) or VEGFR-3 (**D**). Asterisks on each inset reveal carcinoma cell (scale bars on inset, 12.5 mm).

There was no significant difference in the levels of VEGF-C, VEGF-D and VEGFR-3 positivity between samples of benign and borderline tumours (Table 1). The incidence of VEGF-C and VEGF-D detected in ovarian carcinomas was significantly higher than that detected in benign tumours (Table 1, *P*<0.0001, respectively) and borderline tumours (Table 1, *P*=0.0002 and 0.0007, respectively). Similarly, the frequency of VEGFR-3 detected in ovarian carcinomas was significantly higher than that detected in benign tumours (Table 1, *P*<0.0002). VEGF-C and VEGF-D were, in general, homogeneously stained in the cytoplasm of tumour cells in positive cases (Figure 1

). Moreover, they were also expressed in lymphatic and vascular endothelia adjacent to the carcinoma showing strong staining for them (Figure 1). VEGFR-3 was detected in both lymphatic and vascular endothelial cells adjacent to tumour nests in positive cases (Figure 2

). VEGFR-3 staining also highlighted vessel invasion by carcinoma cells (Figure 2).

### Correlation between detection of VEGF-C, VEGF-D and VEGFR-3, and clinico-pathological factors, and relation between detections of VEGF-C or VEGF-D and VEGFR-3 in ovarian carcinomas

When a case with moderate or strong staining was regarded as positive, 39 (66.1%) and 37 (62.7%) of the 59 ovarian carcinomas were positive for VEGF-C and VEGF-D, respectively.

The correlation between the presence of VEGF-C, VEGF-D and VEGFR-3, and clinico-pathological factors in ovarian carcinomas is displayed in Table 2

**Table 2:** Relationship between detection of VEGF-C, VEGF-D and VEGFR-3 and clinico-pathological factors in ovarian carcinoma

**Factors**	**No. of patients**	**No. (%) of VEGF-C positive case**	***P*-value**	**No. (%) of VEGF-D positive case**	***P*-value**	**No. (%) of VEGFR-3 positive case**	***P*-value**
Peritoneal metastasis outside the pelvis							
Negative	39	22(56.4)		21(53.8)		20(51.3)	
Positive	20	17(85.0)	0.03	16(80.0)	0.04	16(80.0)	0.03
							
Lymph node metastasis							
Negative	44	25(56.8)		24(54.5)		21(47.7)	
Positive	15	14(93.3)	0.008	13(86.7)	0.02	15(100)	1E-04
							
Distant metastasis							
Negative	55	35(63.6)		34(61.8)		33(60.0)	
Positive	4	4(100)	NS	3(75.0)	NS	3(75.0)	NS
							
Age							
⩽60	37	22(59.5)		22(59.5)		23(62.1)	
60	22	17(77.3)	NS	15(68.2)	NS	13(59.1)	NS
							
Histology type							
Serous	32	22(68.7)		22(68.7)		21(65.6)	
Nonserous	27	17(63.0)	NS	15(55.6)	NS	15(55.6)	NS
							
Histological grade							
Well	36	22(61.1)		21(58.3)		19(52.8)	
Moderate	15	11(73.3)		10(66.7)		12(80.0)	
Poor	8	6(75.0)	NS	6(75.0)	NS	5(62.5)	NS
							
VEGFR-3							
Negative	23	11(47.8)		10(43.5)		—	
Positive	36	28(77.8)	0.02	27(75.0)	0.02	—	

. The presence of VEGF-C, VEGF-D and VEGFR-3 had no correlation to histological type, grade, distant metastasis or age at surgery. Ovarian carcinomas with lymph node metastasis showed a significantly stronger staining for VEGF-C, VEGF-D and VEGFR-3 than those without (Table 2). Also, the levels of VEGF-C, VEGF-D and VEGFR-3 proteins significantly increased according to the presence of peritoneal metastasis outside the pelvis (Table 2). A significant positive correlation was found between VEGF-C and VEGF-D detected in carcinoma cells and VEGFR-3 detected in endothelial cells adjacent to the carcinoma (Table 2).

### Survival of patients with ovarian carcinomas according to clinico-pathological factors and VEGF-C, VEGF-D or VEGFR-3 detection

We examined carcinoma-specific survival among patients with ovarian carcinoma using univariate analysis according to clinico-pathological factors and the presence of VEGF-C, VEGF-D and VEGFR-3 (Table 3

**Table 3:** Kaplan–Meier survival analysis: Carcinoma-specific survival in ovarian carcinomas according to clinico-pathological factors and VEGF-C, VEGF-D or VEGFR-3 detection

**Variable**	**No. of patients**	**10-year survival rate (%)**	***χ*2**	**Logrank *P*-value**
VEGF-C			3.956	0.046
Negative	20	79.2		
Positive	39	40.1		
				
VEGF-D			9.365	0.002
Negative	22	85.1		
Positive	37	30.2		
				
VEGFR-3			2.282	0.13
Negative	23	69.5		
Positive	36	22.7		
				
Peritoneal metastasis outside the pelvis			23.524	<0.0001
Negative	39	75.2		
Positive	20	Not reached		
				
Lymph node metastasis			25.372	<0.0001
Negative	44	69.1		
Positive	15	9.7		
				
Distant metastasis			1.707	0.19
Negative	55	50		
Positive	4	50		
				
Age			4.736	0.03
⩽60	37	56.2		
60	22	36.1		
				
Histological type			1.789	0.18
Serous	32	31.6		
Nonserous	27	63.3		
				
Histological grade			3.669	0.055
Well	36	69.8		
Moderate and poor	23	Not reached		

). The 10-year survival rate of the 22 patients with VEGF-D-negative carcinoma was 85.1%, whereas that of the 37 patients with VEGF-D-positive carcinoma was 30.2% (Table 3, log-rank, *P*<0.002). While the presence of VEGF-C significantly correlated with poor prognosis (Table 3, log-rank, *P*=0.046), that of VEGFR-3 was not a significant prognostic parameter in ovarian carcinoma (Table 3, log-rank *P*=0.13). Other prognostic factors with significance in univariate analysis were peritoneal metastasis outside the pelvis, lymph node metastasis and age at surgery (Table 3).

We then performed a multivariate regression analysis to determine the independent value of each parameter predicting carcinoma-specific survival (Table 4

**Table 4:** Determination of independent factors affecting survival using Cox regression model

	**Carcinoma-specific survival**		
**Variables**	**Relative risk**	**95% CI**	***P*-value**
VEGF-C			
Negative	1		
Positive	2.7	0.72–37.51	0.101
			
VEGF-D			
Negative	1		
Positive	8.2	2.33–83.33	0.004
			
Peritoneal metastasis outside the pelvis			
Negative	1		
Positive	2.2	0.66–19.61	0.138
			
Lymph node metastasis			
Negative	1		
Positive	5.7	1.44–38.46	0.017
			
Age			
⩽60	1		
60	12.1	2.72–35.71	0.001

). Using Cox regression analysis, we examined prognostic parameters of ovarian carcinomas that were significant in univariate analysis. The presence of VEGF-D was an independent prognostic factor for poor survival (Table 4, relative risk: 8.2, 95% CI: 2.33–83.33). Other independent prognostic factors related to poor survival were lymph node metastasis and age at surgery (Table 4).

## Discussion

We have investigated the distribution of VEGF-C, VEGF-D and VEGFR-3 during progression from a benign tumour to carcinoma. Immunopositivity became more frequent with progression from a benign tumour to carcinoma (Table 1). Each antigen stained more intensely in cases of carcinoma than in benign or borderline tumours (Table 1). Staining was significantly stronger in advanced disease (Table 2). While VEGF-A is involved in tumour progression and maintenance, relatively little is known of the physio-logical role of other members of the VEGF family in tumour progression. Our previous *in situ* hybridisation study suggested that VEGF-B might play a significant role in progression of ovarian tumours (Sowter *et al*, 1997). The present immunohistochemical results suggest a significant role for VEGF-C and VEGF-D in ovarian tumorigenesis. With regard to the association of VEGFR-3 with tumorigenesis, there have been very few reports to date and its contribution to tumour progression is less well understood. While VEGFR-3 expression significantly correlates with tumour progression in breast carcinoma (Gunningham *et al*, 2000), no correlation was found in colon carcinoma (Andre *et al*, 2000). The present immunohistochemical results provide evidence for a possible role for VEGFR-3 in ovarian tumorigenesis.

We have shown that increased levels of VEGF-D protein in ovarian carcinoma are significantly related to peritoneal metastasis outside the pelvis and lymph node metastasis (Table 2). Moreover, increase of VEGF-D protein is independently associated with poor survival in patients with this disease (Table 4). Two recent studies on colorectal carcinoma (CRC) reported contradictory results concerning the relation between VEGF-D detection, clinical parameter and outcome. White *et al*,(2002) showed that high levels of VEGF-D were significantly associated with lymph node involvement and poor survival in CRC, whereas George *et al*,(2001) found significantly lower levels of VEGF-D in CRC than in normal mucosa and no association between the presence of VEGF-D and lymphatic spread. Similarly, low levels of VEGF-D correlate with lymph node metastasis in lung adenocarcinoma (Niki *et al*, 2000), and head and neck squamous cell carcinoma (O-charoenrat *et al*, 2001). However, a significant relation of VEGF-D detection with lymphatic spread has been found in breast carcinoma (Kurebayashi *et al*, 1999). These discrepancies may depend on differences of methodology or antibodies used in each experiment, or may be because of the specific carcinoma explored. Most of these previous studies lacked survival analysis. The present study is the first report to show that VEGF-D is an independent prognostic factor in ovarian carcinomas.

We have also shown a significant association between VEGF-C detection and peritoneal metastasis outside the pelvis as well as lymph node metastasis in ovarian carcinoma (Table 2). In addition, the ovarian carcinoma patients with high levels of VEGF-C protein had a significantly worse prognosis compared to those without (Table 3), although the presence of VEGF-C did not retain significance after multivariate analysis (Table 4). A positive association of VEGF-C protein levels with lymph node metastasis has been found in a variety of carcinomas including stomach, lung, colorectal, oesophageal, prostatic and endometrioid (Tsurusaki *et al*, 1999; Yonemura *et al*, 1999; Akagi *et al*, 2000; Niki *et al*, 2000; Hirai *et al*, 2001; Kajita *et al*, 2001; Kitadai *et al*, 2001). Several previous studies have shown a prognostic aspect of VEGF-C detection in carcinoma tissues. Ichikura *et al*,(2001) reported that high levels of VEGF-C was an independent prognostic determinant in gastric carcinoma, whereas Kajita *et al*,(2001) found no independent impact of VEGF-C detection in nonsmall cell lung carcinoma outcome. Although we could not find prognostic independence of VEGF-C detected in ovarian carcinoma, to the best of our knowledge, this is also the first report documenting a prognostic aspect of VEGF-C expression in this disease.

Recent studies suggest a correlation between the presence of VEGFR-3 and clinical parameters and prognosis in human malignancies. While VEGFR-3 protein levels were not related to lymphatic spread in gastric and breast carcinoma (Yonemura *et al*, 1999; Jacquemier *et al*, 2000), VEGFR-3 detection was found to predict the presence of lymphatic metastases in oral squamous cell carcinoma (Moriyama *et al*, 1997). In the present study, we found a significant correlation between VEGFR-3 expression and lymph node metastasis (Table 2). However, we did not find that outcome was predicted by the presence of VEGFR-3 in endothelial cells adjacent to tumour nests (Table 3), which is consistent with similar studies in breast, nonsmall cell lung and colorectal carcinoma (Jacquemier *et al*, 2000; Kajita *et al*, 2001; White *et al*, 2002).

In the present study, a significant positive correlation was found between VEGF-C or VEGF-D detected in carcinoma cells and VEGFR-3 detected in endothelial cells adjacent to tumour nests (Table 2). A significant association between the levels of detection of VEGF-C and VEGFR-3 has also been found in malignant mesothelioma, gastric carcinoma and nonsmall cell lung carcinoma (Ohta *et al*, 1999; Yonemura *et al*, 1999; Kajita *et al*, 2001), whereas there was no association between VEGF-D and VEGFR-3 detection in colorectal carcinoma (White *et al*, 2002). The present result suggests that VEGF-D and VEGF-C secreted by ovarian carcinoma cells may be able to modulate lymphatic spread and intraperitoneal tumour dissemination through upregulation of VEGFR-3 in endothelial cells of both lymphatic and vascular vessels. We found that VEGFR-3 was expressed not only within lymphatic but also within vascular vessels neighbouring carcinoma nests. VEGFR-3 has been proposed to be a specific marker for lymphatic endothelial cells (Jussila *et al*, 1998; Breiteneder-Geleff *et al*, 1999) and to play a key role in lymphangiogenesis (Jeltsch *et al*, 1997). However, a recent study showed that VEGFR-3 is expressed in the vascular endothelium of breast carcinoma (Valtola *et al*, 1999). Moreover, two more recent studies indicated an association between VEGFR-3 and angiogenesis as well as lymphangiogenesis. Witmer *et al*,(2001) reported that VEGFR-3 was localised in the cytoplasm and on the cell membrane of endothelial cells of sprouting blood vessels and sprouting lymphatic vessels. Niki *et al*,(2001) found that most VEGFR-3 positive vessels detected in invasive lung adenocarcinoma were blood vessels with only a limited number having the characteristics of lymphatic vessels.

Both VEGF-C and VEGF-D were detected not only in carcinoma cells, but also in lymphatic and vascular endothelia adjacent to carcinoma showing strong positive staining (Figure 1). However, they were not detected in vessels distant from the carcinoma. This could simply reflect the expression of VEGF-C and VEGF-D bound to their receptors. Indeed, most of the VEGF-C- and VEGF-D-positive vessels adjacent to ovarian carcinoma examined in this study were also positive for VEGFR-3 (Figure 2). This suggests that they contribute to the regulation of tumour angiogenesis and lymphangiogenesis by establishing a paracrine mechanism in ovarian carcinoma. However, Birner *et al*,(2000) demonstrated that lymphatic microvessel density did not correlate with the progression of epithelial ovarian carcinoma. Thus, although we did not measure microvessel density in this study, it may be necessary in large scale to elucidate a possible role of VEGFR-3 in lymphangiogenesis.

This study clarified that VEGF-C and VEGF-D detected in the carcinoma and VEGFR-3 detected in adjacent endothelial cells have a significant impact on lymph node metastases and intraperitoneal tumour development in ovarian carcinoma. Interestingly, the presence of VEGF-D, but not that of VEGF-C and VEGFR-3, was found to be an independent prognostic factor in ovarian carcinoma. A mechanistic explanation for the prognostic independence of VEGF-D remains unclear. One possible interpretation is that VEGF-D expression is under the control of the c-*fos* proto-oncogene product, which is essential for malignant progression (Orlandini *et al*, 1996). Recently, high expression of VEGF-D has been found to correlate with the upregulation of Fra-1, a member of the AP-1 family of transcription factors (Debinski *et al*, 2001). High levels of AP-1 protein including Fra-1 are related to malignant phenotype and to more advanced stages of disease (Battista *et al*, 1998; Zoumpourlis *et al*, 2000). Collectively, these findings suggest an important role for VEGF-D in transforming the tumour cells into cells with a more malignant nature. However, VEGF-D expression did not correlate with poor-differentiated grade known to possess malignant phenotype in this study. This discrepancy might be explained partly by recent report that gene expression patterns in ovarian carcinomas reflect both morphological features and biological behaviour, regardless of grade using oligonucleotide microarrays (Schwartz *et al*, 2002).

The determination of the presence of VEGF-D in combination with other prognostic factors may enhance the potential to prospectively identify ovarian carcinoma patients who are at risk for poor outcome. Although large-scale studies are necessary to establish the usefulness of VEGF-D expression as a prognostic predictor, VEGF-D may be a promising target for antilymphangiogenic and antiangiogenic therapy in the primary and secondary chemoprevention of ovarian carcinoma.

## References

[bib1] Achen MG, Jeltsch M, Kukk E, Makinen T, Vitali A, Wilks AF, Alitalo K, Stacker SA (1998) Vascular endothelial growth factor D (VEGF-D) is a ligand for the tyrosine kinases VEGF receptor 2 (Flk1) and VEGF receptor 3 (Flt4). Proc Natl Acad Sci USA 95: 548–553 9435229 10.1073/pnas.95.2.548 PMC18457

[bib2] Akagi K, Ikeda Y, Miyazaki M, Abe T, Kinoshita J, Maehara Y, Sugimachi K (2000) Vascular endothelial growth factor-C (VEGF-C) expression in human colorectal cancer tissues. Br J Cancer 83: 887–891 10970690 10.1054/bjoc.2000.1396 PMC2374684

[bib3] Andre T, Kotelevets L, Vaillant JC, Coudray AM, Weber L, Prevot S, Parc R, Gespach C, Chastre E (2000) Vegf, Vegf-B, Vegf-C and their receptors KDR, FLT-1 and FLT-4 during the neoplastic progression of human colonic mucosa. Int J Cancer 86: 174–181 10738243 10.1002/(sici)1097-0215(20000415)86:2<174::aid-ijc5>3.0.co;2-e

[bib4] Baldwin ME, Catimel B, Nice EC, Roufail S, Hall NE, Stenvers KL, Karkkainen MJ, Alitalo K, Stacker SA, Achen MG (2001) The specificity of receptor binding by vascular endothelial growth factor-d is different in mouse and man. J Biol Chem 276: 19166–19171 11279005 10.1074/jbc.M100097200

[bib5] Battista S, de Nigris F, Fedele M, Chiappetta G, Scala S, Vallone D, Pierantoni GM, Mega T, Santoro M, Viglietto G, Verde P, Fusco A, Megar T (1998) Increase in AP-1 activity is a general event in thyroid cell transformation *in vitro* and *in vivo*. Oncogene 17: 377–385 9690519 10.1038/sj.onc.1201953

[bib6] Beck LJ, D'Amore P (1997) Vascular development: cellular and molecular regulation. FASEB J 11: 365–373 9141503

[bib7] Birner P, Schindl M, Obermair A, Plank C, Breitenecker G, Kowalski H, Oberhuber G (2000) Lymphatic microvessel density in epithelial ovarian cancer: its impact on prognosis. Anticancer Res 20: 2981–2985 11062711

[bib8] Breiteneder-Geleff S, Soleiman A, Kowalski H, Horvat R, Amann G, Kriehuber E, Diem K, Weninger W, Tschachler E, Alitalo K, Kerjaschki D (1999) Angiosarcomas express mixed endothelial phenotypes of blood and lymphatic capillaries: podoplanin as a specific marker for lymphatic endothelium. Am J Pathol 154: 385–394 10027397 10.1016/S0002-9440(10)65285-6 PMC1849992

[bib9] Cao Y, Linden P, Farnebo J, Cao R, Eriksson A, Kumar V, Qi JH, Claesson-Welsh L, Alitalo K (1998) Vascular endothelial growth factor C induces angiogenesis *in vivo*. Proc Natl Acad Sci USA 95: 14389–14394 9826710 10.1073/pnas.95.24.14389 PMC24383

[bib10] Debinski W, Slagle-Webb B, Achen MG, Stacker SA, Tulchinsky E, Gillespie GY, Gibo DM (2001) VEGF-D is an X-linked/AP-1 regulated putative onco-angiogen in human glioblastoma multiform. Mol Med 7: 598–608 11778649 PMC1950071

[bib11] Ellis L, Fidler I (1996) Angiogenesis and metastasis. Eur J Cancer 32A: 2451–2460 9059333 10.1016/s0959-8049(96)00389-9

[bib12] Folkman J (1990) What is the evidence that tumors are angiogenesis dependent? J Natl Cancer Inst 82: 4–6 1688381 10.1093/jnci/82.1.4

[bib13] Folkman J (1995) Angiogenesis in cancer, vascular, rheumatoid and other disease. Nat Med 1: 27–31 7584949 10.1038/nm0195-27

[bib14] George ML, Tutton MG, Janssen F, Arnaout A, Abulafi AM, Eccles SA, Swift RI (2001) VEGF-A, VEGF-C, and VEGF-D in colorectal cancer progression. Neoplasia 3: 420–427 11687953 10.1038/sj.neo.7900186 PMC1506210

[bib15] Greenlee RT, Murray T, Bolden S, Wingo PA (2000) Cancer statistics. Cancer J Clin 50: 7–33 10.3322/canjclin.50.1.7 10735013

[bib16] Gunningham SP, Currie MJ, Han C, Robinson BA, Scott PA, Harris AL, Fox SB (2000) The short form of the alternatively spliced flt-4 but not its ligand vascular endothelial growth factor C is related to lymph node metastasis in human breast cancers. Clin Cancer Res 6: 4278–4286 11106244

[bib17] Hirai M, Nakagawara A, Oosaki T, Hayashi Y, Hirono M, Yoshihara T (2001) Expression of vascular endothelial growth factor (VEGF-A/VEGF-1 and VEGF-C/VEGF-2) in postmenopausal uterine endometrial carcinoma. Gynecol Oncol 80: 181–188 11161857 10.1006/gyno.2000.6056

[bib18] Ichikura T, Tomimatsu S, Ohkura E, Mochizuki H (2001) Prognostic significance of the expression of vascular endothelial growth factor (VEGF) and VEGF-C in gastric carcinoma. J Surg Oncol 78: 132–137 11579392 10.1002/jso.1133

[bib19] Jacquemier J, Mathoulin-Portier MP, Valtola R, Charafe-Jauffret E, Geneix J, Houvenaeghel G, Puig B, Bardou VJ, Hassoun J, Viens P, Birnbaum D (2000) Prognosis of breast-carcinoma lymphagenesis evaluated by immunohistochemical investigation of vascular-endothelial-growth-factor receptor 3. Int J Cancer 89: 69–73 10719733 10.1002/(sici)1097-0215(20000120)89:1<69::aid-ijc11>3.0.co;2-m

[bib20] Jeltsch M, Kaipainen A, Joukov V, Meng X, Lakso M, Rauvala H, Swartz M, Fukumura D, Jain RK, Alitalo K (1997) Hyperplasia of lymphatic vessels in VEGF-C transgenic mice. Science 276: 1423–1425 9162011 10.1126/science.276.5317.1423

[bib21] Joukov V, Pajusola K, Kaipainen A, Chilov D, Lahtinen I, Kukk E, Saksela O, Kalkkinen N, Alitalo K (1996) A novel vascular endothelial growth factor, VEGF-C, is a ligand for the Flt4 (VEGFR-3) and KDR (VEGFR-2) receptor tyrosine kinases. EMBO J 15: 290–298 8617204 PMC449944

[bib22] Joukov V, Sorsa T, Kumar V, Jeltsch M, Claesson-Welsh L, Cao Y, Saksela O, Kalkkinen N, Alitalo K (1997) Proteolytic processing regulates receptor specificity and activity of VEGF-C. EMBO J 16: 3898–3911 9233800 10.1093/emboj/16.13.3898 PMC1170014

[bib23] Jussila L, Valtola R, Partanen TA, Salven P, Heikkila P, Matikainen MT, Renkonen R, Kaipainen A, Detmar M, Tschachler E, Alitalo R, Alitalo K (1998) Lymphatic endothelium and Kaposi's sarcoma spindle cells detected by antibodies against the vascular endothelial growth factor receptor-3. Cancer Res 58: 1599–1604 9563467

[bib24] Kaipainen A, Korhonen J, Mustonen T, van Hinsbergh VW, Fang GH, Dumont D, Breitman M, Alitalo K (1995) Expression of the fms-like tyrosine kinase 4 gene becomes restricted to lymphatic endothelium during development. Proc Natl Acad Sci USA 92: 3566–3570 7724599 10.1073/pnas.92.8.3566 PMC42208

[bib25] Kajita T, Ohta Y, Kimura K, Tamura M, Tanaka Y, Tsunezuka Y, Oda M, Sasaki T, Watanabe G (2001) The expression of vascular endothelial growth factor C and its receptors in non-small cell lung cancer. Br J Cancer 85: 255–260 11461086 10.1054/bjoc.2001.1882 PMC2364042

[bib26] Kitadai Y, Amioka T, Haruma K, Tanaka S, Yoshihara M, Sumii K, Matsutani N, Yasui W, Chayama K (2001) Clinicopathological significance of vascular endothelial growth factor (VEGF)-C in human esophageal cell carcinomas. Int J Cancer 93: 662–666 11477575 10.1002/ijc.1379

[bib27] Kurebayashi J, Otsuki T, Kunisue H, Mikami Y, Tanaka K, Yamamoto S, Sonoo H (1999) Expression of vascular endothelial growth factor (VEGF) family members in breast cancer. Jpn J Cancer Res 90: 977–981 10551327 10.1111/j.1349-7006.1999.tb00844.x PMC5926164

[bib28] Makinen T, Jussila L, Veikkalo T, Karpanen T, Kettunen MI, Pulkkanen KJ, Kauppinen R, Jackson DG, Kubo H, Nishikawa S, Yla-Herttuala S, Alitalo K (2001) Inhibition of lymphangiogenesis with resulting lymphedema in transgenic mice expressing soluble VEGF receptor-3. Nat Med 7: 199–205 11175851 10.1038/84651

[bib29] Mandriota SJ, Jussila L, Jeltsch M, Compagni A, Baetens D, Prevo R, Banerji S, Huarte J, Montesano R, Jackson DG, Orci L, Alitalo K, Christofori G, Pepper MS (2001) Vascular endothelial growth factor-C-mediated lymphangiogenesis promotes tumour metastasis. EMBO J 20: 672–682 11179212 10.1093/emboj/20.4.672 PMC145430

[bib30] Moriyama M, Kumagai S, Kawashiri S, Kojima K, Kakihara K, Yamamoto E (1997) Immunohistochemical study of tumor angiogenesis in oral squamous cell carcinoma. Oral Oncol 33: 369–374 9415339 10.1016/s1368-8375(97)00025-0

[bib31] Neufeld G, Cohen T, Gengrinovitch S, Poltorak Z (1999) Vascular endothelial growth factor (VEGF) and its receptors. FASEB J 13: 9–22 9872925

[bib32] Nicosia RF (1998) What is the role of vascular endothelial growth factor-related molecules in tumor angiogenesis? Am J Pathol 153: 11–16 9665459 10.1016/S0002-9440(10)65539-3 PMC1852942

[bib33] Niki T, Iba S, Tokunou M, Yamada T, Matsuno Y, Hirohashi S (2000) Expression of vascular endothelial growth factor A, B, C, and D and their relationships to lymph node status in lung adenocarcinoma. Clin Cancer Res 6: 2431–2439 10873096

[bib34] Niki T, Iba S, Yamada T, Matsuno Y, Enholm B, Hirohashi S (2001) Expression of vascular endothelial growth factor receptor 3 in blood and lymphatic vessels of lung adenocarcinoma. J Pathol 193: 450–457 11276003 10.1002/path.828

[bib35] O-charoenrat P, Rhys-Evans P, Eccles SA (2001) Expression of vascular endothelial growth factor family members in head and neck squamous cell carcinoma correlates with lymph node metastasis. Cancer 92: 556–568 11505400 10.1002/1097-0142(20010801)92:3<556::aid-cncr1355>3.0.co;2-q

[bib36] Ohta Y, Shridhar V, Bright RK, Kalemkerian GP, Du W, Carbone M, Watanabe Y, Pass HI (1999) VEGF and VEGF type C play an important role in angiogenesis and lymphangiogenesis in human malignant mesothelioma tumours. Br J Cancer 81: 54–61 10487612 10.1038/sj.bjc.6690650 PMC2374345

[bib37] Orlandini M, Marconcini L, Ferruzzi R, Oliviero S (1996) Identification of a c-fos-induced gene that is related to the platelet-derived growth factor/vascular endothelial growth factor family. Proc Natl Acad Sci USA 93: 11675–11680 8876195 10.1073/pnas.93.21.11675 PMC38117

[bib38] Pepper MS, Mandriota SJ, Jeltsch M, Kumar V, Alitalo K (1998) Vascular endothelial growth factor (VEGF)-C synergizes with basic fibroblast growth factor and VEGF in the induction of angiogenesis *in vitro* and alters endothelial cell extracellular proteolytic activity. J Cell Physiol 177: 439–452 9808152 10.1002/(SICI)1097-4652(199812)177:3<439::AID-JCP7>3.0.CO;2-2

[bib39] Schwartz DR, Kardia SL, Shedden KA, Kuick R, Michailidis G, Taylor JM, Misek DE, Wu R, Zhai Y, Darrah CM, Reed H, Ellenson LH, Giordano TJ, Fearon ER, Hanash SM, Cho KR (2002) Gene expression in ovarian cancer reflects both morphology and biological behavior, distinguishing clear cell from other poor-prognosis ovarian carcinomas. Cancer Res 62: 4722–4729 12183431

[bib40] Skobe M, Hawighorst T, Jackson DG, Prevo R, Janes L, Velasco P, Riccardi L, Alitalo K, Claffey K, Detmar M (2001) Induction of tumor lymphangiogenesis by VEGF-C promotes breast cancer metastasis. Nat Med 7: 192–198 11175850 10.1038/84643

[bib41] Sowter HM, Corps AN, Evans AL, Clark DE, Charnock-Jones DS, Smith SK (1997) Expression and localization of the vascular endothelial growth factor family in ovarian epithelial tumors. Lab Invest 77: 607–614 9426398

[bib42] Stacker SA, Caesar C, Baldwin ME, Thornton GE, Williams RA, Prevo R, Jackson DG, Nishikawa S, Kubo H, Achen MG (2001) VEGF-D promotes the metastatic spread of tumor cells via the lymphatics. Nat Med 7: 186–191 11175849 10.1038/84635

[bib43] Tsurusaki T, Kanda S, Sakai H, Kanetake H, Saito Y, Alitalo K, Koji T (1999) Vascular endothelial growth factor-C expression in human prostatic carcinoma and its relationship to lymph node metastasis. Br J Cancer 80: 309–313 10390013 10.1038/sj.bjc.6690356 PMC2362987

[bib44] Umemoto M, Yokoyama Y, Sato S, Tsuchida S, Al-Mulla F, Saito Y (2001) Carbonyl reductase as a significant predictor of survival and lymph node metastasis in epithelial ovarian cancer. Br J Cancer 85: 1032–1036 11592776 10.1054/bjoc.2001.2034 PMC2375107

[bib45] Valtola R, Salven P, Heikkila P, Taipale J, Joensuu H, Rehn M, Pihlajaniemi T, Weich H, deWaal R, Alitalo K (1999) VEGFR-3 and its ligand VEGF-C are associated with angiogenesis in breast cancer. Am J Pathol 154: 1381–1390 10329591 10.1016/S0002-9440(10)65392-8 PMC1866582

[bib46] White JD, Hewett PW, Kosuge D, McCulloch T, Enholm BC, Carmichael J, Murray JC (2002) Vascular endothelial growth factor-D expression is an independent prognostic marker for survival in colorectal carcinoma. Cancer Res 62: 1669–1675 11912138

[bib47] Witmer AN, van Blijswijk BC, Dai J, Hofman P, Partanen TA, Vrensen GFJM, Schlingemann RO (2001) VEGFR-3 in adult angiogenesis. J Pathol 195: 490–497 11745682 10.1002/path.969

[bib48] Witzenbichler B, Asahara T, Murohara T, Silver M, Spyridopoulos I, Magner M, Principe N, Kearney M, Hu JS, Isner JM (1998) Vascular endothelial growth factor-C (VEGF-C/VEGF-2) promotes angiogenesis in the setting of tissue ischemia. Am J Pathol 153: 381–394 9708799 10.1016/S0002-9440(10)65582-4 PMC1852989

[bib49] Yokoyama Y, Sakamoto T, Sato S, Saito Y (1999) Evaluation of cytoreductive surgery with pelvic and paraaortic lymphadenectomy and intermittent cisplatin-based combination chemotherapy for improvement of long-term survival in ovarian cancer. Eur J Gynaecol Oncol 20: 361–366 10609495

[bib50] Yokoyama Y, Sato S, Futagami M, Fukushi Y, Sakamoto T, Umemoto M, Saito Y (2000) Prognostic significance of vascular endothelial growth factor and its receptors in endometrial carcinoma. Gynecol Oncol 77: 413–418 10831352 10.1006/gyno.2000.5802

[bib51] Yonemura Y, Endo Y, Fujita H, Fushida S, Ninomiya I, Bandou E, Taniguchi K, Miwa K, Ohoyama S, Sugiyama K, Sasaki T (1999) Role of vascular endothelial growth factor C expression in the development of lymph node metastasis in gastric cancer. Clin Cancer Res 5: 1823–1829 10430087

[bib52] Zoumpourlis V, Papassava P, Linardopoulos S, Gillespie D, Balmain A, Pintzas A (2000) High levels of phosphorylated c-Jun, Fra-1, Fra-2 and ATF-2 proteins correlate with malignant phenotypes in the multistage mouse skin carcinogenesis model. Oncogene 19: 4011–4021 10962557 10.1038/sj.onc.1203732

